# Construction of functional brain connectivity networks from fMRI data with driving and modulatory inputs: an extended conditional Granger causality approach

**DOI:** 10.3934/Neuroscience.2020005

**Published:** 2020-04-10

**Authors:** Evangelos Almpanis, Constantinos Siettos

**Affiliations:** 1 Section of Condensed Matter Physics, National and Kapodistrian University of Athens, Greece; 2 Institute of Nanoscience and Nanotechnology, NCSR “Demokritos,” Athens, Greece; 3 Dipartimento di Matematica e Applicazioni “Renato Caccioppoli”, Università degli Studi di Napoli Federico II, Italy

**Keywords:** functional connectivity networks, data-based analysis, Granger Causality, task fMRI, stimuli and modulatory inputs, dynamical causal modelling

## Abstract

We propose a numerical-based approach extending the conditional MVAR Granger causality (MVGC) analysis for the construction of directed connectivity networks in the presence of both exogenous/stimuli and modulatory inputs. The performance of the proposed scheme is validated using both synthetic stochastic data considering also the influence of haemodynamics latencies and a benchmark fMRI dataset related to the role of attention in the perception of visual motion. The particular fMRI dataset has been used in many studies to evaluate alternative model hypotheses using the Dynamic Causal Modelling (DCM) approach. Based on the use of the Bayes factor, we show that the obtained GC connectivity network compares well to a reference model that has been selected through DCM analysis among other candidate models. Thus, our findings suggest that the proposed scheme can be successfully used as a stand-alone or complementary to DCM approach to find directed causal connectivity patterns in task-related fMRI studies.

## 1. Introduction

Unfolding the complexity of the human brain function, as this is related to brain networking during cognitive tasks and/or at resting state, is one of the most significant and challenging research pursuits of our time. Depending on the questions asked, different connectivity *modes* may be sought [1]. For example, functional connectivity analysis seeks for statistical dynamic inter-dependencies between time series, while effective connectivity analysis seeks for causal-mechanistic influences that neural units exert to each other. On the other hand, structural connectivity seeks for anatomical connections between neuronal regions. Nevertheless, in the literature, a debate still exists considering the differences between effective and functional connectivity, and as a result, multiple definitions exist. In this work we follow the definition stated in Refs. [2], [3]. Towards the reconstruction of brain connectivity, various data-driven and model-driven approaches for *mining* the useful information included in datasets acquired by neuroimaging techniques such as Electroencephalography (EEG), Magnetoencephalography (MEG), Functional Magnetic Resonance Imaging (fMRI) and Positron Emission Tomography (PET) have been proposed (for a review see also [4], [5]). These extend from simple non-parametric methods such as correlation and coherence [6]–[9] to mutual information [10]–[13] and phase synchronization methods [14]–[17] and from linear data reduction methods such as Principal Component Analysis (PCA) and Independent Component Analysis (ICA) [18]–[20] to non-linear manifold learning methods such as ISOMAP [21], [22] and Diffusion Maps [23]. Parametric approaches include Granger-causality-based methods [24]–[29] and Dynamic Causal Modeling (DCM) for modelling the effective brain connectivity [30]. Relatively simple statistical approaches such as correlation and coherence analysis continue to hold a significant share in the modeling of brain (functional) connectivity of brain regions (see e.g. [8], [9]) especially on the basis of fMRI data [31], [32], however, these approaches fail in general to detect directional and multivariate dependencies. On the other hand, the concept of causality as introduced by Norbert Wiener [33] and formalized by Clive Granger [24] in economics has triggered significant further developments in neuroscience. According to the simplest form of Granger-causality (GC) a time-series *X* drives/influences another time series *Y* if past values of *X* help forecast better the evolution of *Y* as compared to using just the past values of *Y*. In its more general form, GC is performed on the basis of multivariate linear regression models. It has been applied in several studies, mainly in EEG and MEG [34]–[41]) but also in fMRI studies (see e.g. [42]–[53]).

GC is mainly a data-driven modeling approach for accessing functional connectivity that does not require any prior knowledge about the brain areas involved in a specific cognitive task. The (directed) interconnections between brain regions emerge from the statistical inter-dependencies between the corresponding emerged brain signals. On the the other hand, Dynamic Causal Modeling (DCM) is the most known representative method for reconstructing the underlying task-dependent effective connectivity from fMRI data and it is mainly model-driven [30], [54], [55]. In particular, it requires a prior knowledge (or guess) of the specific brain areas that are involved in the function of a specific cognitive task. DCM compares the efficiency of the various models (brain interconnections) under a Bayesian-based comparison to decide which model is the best in fitting the observed fMRI data. One of the advantages of DCM is that it inherently incorporates the experimental driving stimuli and modulatory inputs. For a a comparative evaluation and review of GC and DCM see Friston et al. [3].

Although GC could be in principle extended to find statistical dependencies from driving inputs (i.e. using multivariate autoregressive models with exogenous inputs (MVARX)) and furthermore identify modulatory effects on the functional connectivity network, only a very few number of studies have exploited this possibility (and even fewer in real experimental data). For example, Guo et al. [56] introduced partial GC and showed that partial GC cannot completely eliminate the influence of exogenous inputs and latent variables in all cases and that a complete elimination is only possible if “all common inputs have equal influence on all measured variables”. In their analysis, they considered various toy models with common exogenous inputs. They also considered the modelling of local field potential (LFP) data collected from the inferotemporal cortex of sheep; the (visual) stimulus signal was common to all electrodes. Roelstraete et al. [57] examined the efficiency of partial GC in the presence of none, weak and strong exogenous and latent variables. They concluded that in the presence of unknown latent and exogenous influences, the partial GC performs better than the conditional GC. The above studies examined the impact of exogenous inputs and latent influences on the efficiency of partial and conditional Granger, but they did not considered their identification/integration into the functional connectivity network. Alternatively, Ge et al. [58] proposed an extension of GC to handle exogenous inputs using a bilinear approximation similar to that of the DCM, in an attempt to incorporate the influence of external/experimental inputs. For the implementation of their approach, similarly to the DCM implementation, an a-priori knowledge of the specific regions that are driven by the input and/or the connections that are being modulated is assumed. In this respect, the development of extended GC schemes and the examination of their efficiency to deal with exogenous driving inputs and to identify the modulatory effects as those arise in real fMRI experiments is still an open problem. Towards this aim, Bajaj et al. [59] showed that non-parametric GC for pairwise calculations [60] and DCM give similar qualitative and quantitative results in the context of both simulated and real resting state fMRI.

In this work, we address an extended conditional MVGC modelling approach, which, apart from the *emergent* functional connectivity network reflecting the task-related brain organization, it identifies both the prominent stimulated regions driven by external stimuli and assess the impact of the modulation to the emerged interconnections. First, we present the extended GC modelling approach, and then we employ it to reconstruct the functional connectivity network based on stochastic synthetic data that involves external stimuli and modulation. Furthermore, we examine the impact of the haemodynamics in the inferred connectivity scheme. We then apply the proposed scheme to reconstruct the emergent functional connectivity network from a real fMRI experiment designed to investigate the role of the attention in the perception of the visual motion. This is a benchmark problem (see e.g. Ref. [59]) that has been exploited to validate various models within the DCM framework. We show that the proposed GC scheme results to a consistent connectivity pattern that compares well with the reference/best model obtained with the DCM approach and we also assess the computational cost of the methods.

## 2. Functional connectivity under an extended conditional Granger Causality approach

The proposed methodology for the construction of the complete causality network that contains exogenous stimuli and modulatory inputs follows a three-tiered approach. In particular, in the first stage, we identify the *emergent* functional connectivity network using a multivariate (conditional) Granger causality (MVGC) analysis, without taking into account in the model, the presence of the exogenous and the modulatory inputs. At this point we should note that the *emergent* functional networks are different from the *intrinsic* cognitive networks i.e. the resting state networks (RSNs) [61], [62] that pertain to the activity of the brain in the absence of an external imposed task. When external stimuli are present, then the *emergent* (functional) connectivity network between brain regions is task-related and thus differs from the *intrinsic* resting-state activity. Hence, the time series reflecting the activity of the brain regions of the *emergent* task-related network will be more or less affected by the exogenous and modulatory inputs. However, it is expected that the exogenous/stimuli and modulatory input(s) will be more prominent, i.e. it will influence more the activity of particular region(s) and interconnections and that this will be reflected on larger values of causalities.

In the second stage, after the identification of the *emergent* functional connectivity network, we assess the influence that the exogenous input exerts on each one of the brain regions through a pairwise GC analysis. As discussed above, the hypothesis is that the exogenous inputs will affect more only one of the activated brain regions.

Finally in the third stage, the effect of modulatory inputs is assessed by formulating and solving an augmented MVGC problem. In the following sections we describe in detail the proposed approach.

### 2.1. Emergent functional brain connectivity: pairwise and conditional MVGC analysis

In this section, we review the concepts of pairwise and conditional MVGC analysis for the construction of functional connectivity networks that are used for the construction of the *emergent* network as discussed above.

GC was initially introduced in 1969 by Clive Granger as a linear regression formalism of the idea of Wiener and Akaike [33] that a time series *Y_t_*^(2)^ causes/drives another time series *Y_t_*^(1)^, if the knowledge of *Y_t_*^(2)^ helps to predict the future evolution of *Y_t_*^(1)^ [25], [27], [28]. According to GC, the casual flow from a time series *Y_t_*^(2)^ to another time series *Y_t_*^(1)^ can be quantified by comparing two linear auto-regressive (AR) models for *Y_t_*^(1)^: one containing only previous observations of *Y_t_*^(1)^ (restricted model), and one containing previous observations of both *Y_t_*^(1)^ and *Y_t_*^(2)^ (full model). If the prediction error in the full model is smaller compared to that of the restricted model, then we state that *Y_t_*^(2)^ *causes* *Y_t_*^(1)^. The full linear AR model that is driven by past instances of both time series *Y_t_*^(1)^ and *Y_t_*^(2)^ can be written as:

$$Y_{t}^{\left(1\right)}=\sum_{j=1}^{p}A_{j}^{\left(1,1\right)}Y_{t-j}^{\left(1\right)}+\sum_{j=1}^{p}A_{j}^{\left(1,2\right)}Y_{t-j}^{\left(2\right)}+e_{t}^{\left(1\right)}$$ (1)

$$Y_{t}^{\left(2\right)}=\sum_{j=1}^{p}A_{j}^{\left(2,1\right)}Y_{t-j}^{\left(1\right)}+\sum_{j=1}^{p}A_{j}^{\left(2,2\right)}Y_{t-j}^{\left(2\right)}+e_{t}^{\left(2\right)},$$ (2)

while the restricted AR models read:

$$Y_{t}^{\left(1\right)}=\sum_{j=1}^{p}{A^{\prime }}_{j}^{\left(1,1\right)}Y_{t-j}^{\left(1\right)}+{e^{\prime }}_{t}^{\left(1\right)},$$ (3)

$$Y_{t}^{\left(2\right)}=\sum_{j=1}^{p}{A^{\prime }}_{j}^{\left(2,2\right)}Y_{t-j}^{\left(2\right)}+{e^{\prime }}_{t}^{\left(2\right)},$$ (4)

in which *Y_t_*^(*i*)^, *i* = 1,2 depend only on their own past; $A_{j}^{\left(1,1\right)}$, $A_{j}^{\left(1,2\right)}$, $A_{j}^{\left(2,1\right)}$, $A_{j}^{\left(2,2\right)}$, ${A^{\prime }}_{j}^{\left(1,1\right)}$, ${A^{\prime }}_{j}^{\left(2,2\right)}$ are the regression coefficients, and $e_{t}^{\left(i\right)},{e^{\prime }}_{t}^{\left(i\right)},i=1,2$ are the corresponding i.i.d. residuals with zero mean reflecting the prediction errors. For the application of the above AR models it is necessary for the stochastic time series to be stationary. Most of the times, the weak stationarity assumption is required meaning that the first (mean value) and second (variance) moments of the time series distribution are time invariant and that the covariance is also time invariant depending only on the selection of the time-lag.

The magnitude of the GC that quantifies whether or not the full AR model is better than the restricted AR model in predicting the temporal evolution of the time series (i.e. that *Y_t_*^(*k*)^ *causes Y_t_*^(*l*)^), (*k*, *l* = 1,2, *k* ≠ *l*) is defined as [26]:

$$F_{Y^{\left(k\right)}\to Y^{\left(l\right)}}=ln\frac{\left|\Sigma^{\prime }\right|}{\left|\Sigma \right|}$$ (5)

where $\Sigma =var(e_{t}^{\left(l\right)})$ and $\Sigma^{\prime }=var({e^{\prime }}_{t}^{\left(l\right)})$ are the estimators of the variances of the residuals of the full (Eq. 1 or Eq. 2) and the restricted model (Eq. 3 or Eq. 4), respectively.

Note that if $\left|\Sigma^{\prime }\right|=\left|\Sigma \right|$, $F_{Y^{\left(k\right)}\to Y^{\left(l\right)}}=0$, meaning that the addition of *Y*^(*k*)^ into the regression model does not result into a reduction of the variance, thus *Y*^(*k*)^ does not influence *Y*^(*l*)^.

Thus, GC tests the null hypothesis:

$$H_{0}:A_{1}^{\left(k,k\right)}=A_{2}^{\left(k,k\right)}=\ldots =A_{p}^{\left(k,k\right)}=0,$$ (6)

given that the time series are stationary. It can be shown that under certain assumptions [63], the above GC measure follows a *χ*^2^ distribution.

**Figure 1.**
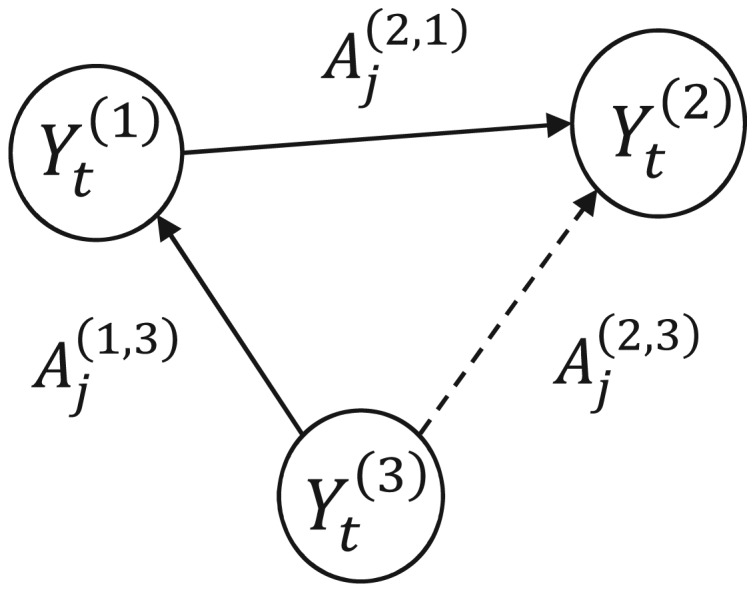
A schematic of three interconnected groups of time series. The pairwise GC may provide spurious connections.

In cases of indirect-joint influences between (groups of) more than two time series, the above “direct” pairwise-based formulation will provide spurious connections (see e.g. [60]). Take for example the paradigm of three groups of time series connected as shown in Figure 1, where the (group of) time series *Y_t_*^(3)^ influences *Y_t_*^(1)^ and consequently *Y_t_*^(1)^ influences *Y_t_*^(2)^. The pairwise GC may result to a statistically significant causality from *Y_t_*^(3)^ to *Y_t_*^(2)^.

Thus, when more than two regions are getting involved, it is required to access the conditional/multivariate GC (MVGC) [26], [28], [60], [63], [64].

Let us consider *N* (groups of) time series, and $Y_{t}=\left\{Y_{t}^{\left(1\right)},Y_{t}^{\left(2\right)},\ldots Y_{t}^{\left(N\right)}\right\}$ the vector containing the *t*-th sample of the *N*-dimensional time series *Y_t_^i^*, *i* = 1, … *N*. A multivariate AR (MVAR) model of order *p* can then be written as:

$$Y_{t}=\sum_{i=1}^{p}Y_{t-i}A_{i}+e_{t},$$ (7)

where **A_i_** is a *N* × *N* matrix containing the regression coefficients of the MVAR model and $e_{t}=\left\{e_{t}^{\left(1\right)},e_{t}^{\left(2\right)},\ldots e_{t}^{\left(N\right)}\right\}$ is the vector of the residuals of the MVAR model at time *t*; the residuals are assumed to be i.i.d. variables with zero mean. The (sample) covariance matrix of the residuals reads:

$$\sum_{ee}=\frac{1}{N}\sum_{t=1}^{N}e_{t}e_{t}{}^{T}$$ (8)

For example for three (groups of) time series **Y**^(*l*)^, **Y**^(*k*)^, **Y**^(*m*)^ the MVAR model reads:

$$Y_{t}^{\left(l\right)}=\sum_{j=1}^{p}A_{j}^{\left(l,l\right)}Y_{t-j}^{\left(l\right)}+\sum_{j=1}^{p}A_{j}^{\left(l,k\right)}Y_{t-j}^{\left(k\right)}+\sum_{j=1}^{p}A_{j}^{\left(l,m\right)}Y_{t-j}^{\left(m\right)}++e_{t}^{\left(l\right)}$$ (9)

$$Y_{t}^{\left(k\right)}=\sum_{j=1}^{p}A_{j}^{\left(k,l\right)}Y_{t-j}^{\left(l\right)}+\sum_{j=1}^{p}A_{j}^{\left(k,k\right)}Y_{t-j}^{\left(k\right)}+\sum_{j=1}^{p}A_{j}^{\left(k,m\right)}Y_{t-j}^{\left(m\right)}++e_{t}^{\left(k\right)}$$ (10)

$$Y_{t}^{\left(m\right)}=\sum_{j=1}^{p}A_{j}^{\left(m,l\right)}Y_{t-j}^{\left(l\right)}+\sum_{j=1}^{p}A_{j}^{\left(m,k\right)}Y_{t-j}^{\left(k\right)}+\sum_{j=1}^{p}A_{j}^{\left(m,m\right)}Y_{t-j}^{\left(m\right)}++e_{t}^{\left(m\right)}$$ (11)

The covariance matrix ∑*_M_* of the residual/ noise vectors **e***_t_*^(*l*)^,**e***_t_*^(*k*)^,**e***_t_*^(*m*)^ is given by:

$$\sum_{M}=\left(\begin{array}{ccc} \sum_{e^{l}e^{l}} & \sum_{e^{l}e^{k}} & \sum_{e^{l}e^{m}}\\ \sum_{\varepsilon^{l}e^{l}} & \sum_{e^{l}e^{k}} & \sum_{e^{l}e^{m}}\\ \sum_{e^{l}e^{l}} & \sum_{e^{l}\varepsilon^{k}} & \sum_{e^{l}e^{m}} \end{array}\right)$$ (12)

According to the above formulation, the conditional GC measure from **Y**^(*k*)^ to **Y**^(*l*)^ given/conditioned in (i.e. in the presence of) **Y**^(*m*)^ is given by:

$$F_{Y^{\left(k\right)}\to Y^{\left(l\right)}|Y^{\left(m\right)}}=ln\frac{\left|{\sum^{\prime }}_{M}\right|}{\left|\sum_{\varepsilon^{l}\varepsilon^{l}}\right|},$$ (13)

and, ${\sum^{\prime }}_{M}$ is the covariance of the residual vector **e′**^(*l*)^ resulting from the restricted (sub) model:

$$Y_{t}^{\left(l\right)}=\sum_{j=1}^{p}{A^{\prime }}_{j}^{\left(l,l\right)}Y_{t-j}^{\left(l\right)}+\sum_{j=1}^{p}{A^{\prime }}_{j}^{\left(l,m\right)}Y_{t-j}^{\left(m\right)}+{e^{\prime }}_{t}^{\left(l\right)}.$$ (14)

### 2.2. Identification of externally-driven brain regions and modulated inter-connections: an extended conditional MVGC approach

Here, we extend the above MVGC analysis, by considering an MVARX modeling approach in order to include exogenous inputs and assess the modulatory effects on the connectivity patterns. The first step of the approach is the reconstruction of the *emergent* connectivity (i.e. the functional connectivity resulting by excluding the exogenous inputs and modulations) based on the conventional MVGC analysis. In the second step, the scheme quantifies the influence of exogenous driving inputs (for example an experimentally designed visual or auditory stimulation) on each particular brain region involved in the given emergent connectivity network as computed in the first stage. Finally, the scheme access whether and where an exogenous modulatory signal influences the interconnections.

#### 2.2.1. MVARX modelling of exogenous driving inputs

To begin with, we assume a deterministic known (experimentally designed) exogenous stimulus *u*(*t*) that acts on (excites) a particular brain region, out of *n* *emergent* inter-connected regions. The biophysical assumption here is that, the GC is directed from the exogenous driving signal to a particular brain region (which has to be identified). Let us inductively present the modelling approach by taking the time series **Y***_t_* = {*Y*^(1)^,*Y*^(2)^,…*Y*^(*n*)^}∈*R^n^* representing signals from *n* brain regions, while, at the same time, an exogenous input (a stimulus) *u*(*t*) is presented, though we do not know which brain region(s) this stimulus excites. We also assume that we have identified by MVGC the *emergent* functional connectivity network, i.e. we have constructed an MVAR model given by Eq. (9)–(11). In order to identify which one of the regions the exogenous input affects more, we calculate the *n* corresponding pairwise GCs, namely $F_{u\to Y^{\left(i\right)}}$, *i* = 1,2,…*n* based on the following MVARX model:

$$Y_{t}=\sum_{i=1}^{p}Y_{t-i}{\hat{A}}_{i}+\sum_{i=1}^{q}B_{i}u_{t-i}+\varepsilon_{t},$$ (15)

For demonstration purposes, without the loss of generality, let us consider again three (groups of) time series **Y**^(*l*)^, **Y**^(*k*)^, **Y**^(*m*)^. Then the corresponding MVARX model reads:

$$Y_{t}^{\left(l\right)}=\sum_{j=1}^{p}{\hat{A}}_{j}^{\left(l,l\right)}Y_{t-j}^{\left(l\right)}+\sum_{j=1}^{p}{\hat{A}}_{j}^{\left(l,k\right)}Y_{t-j}^{\left(k\right)}+\sum_{j=1}^{p}{\hat{A}}_{j}^{\left(l,m\right)}Y_{t-j}^{\left(m\right)}+\sum_{i=1}^{q}B_{i}^{\left(l\right)}u_{t-i}+\varepsilon_{t}^{\left(l\right)}$$ (16)

$$Y_{t}^{\left(k\right)}=\sum_{j=1}^{p}{\hat{A}}_{j}^{\left(k,l\right)}Y_{t-j}^{\left(l\right)}+\sum_{j=1}^{p}{\hat{A}}_{j}^{\left(k,k\right)}Y_{t-j}^{\left(k\right)}+\sum_{j=1}^{p}{\hat{A}}_{j}^{\left(k,m\right)}Y_{t-j}^{\left(m\right)}+\sum_{i=1}^{q}B_{i}^{\left(k\right)}u_{t-i}+\varepsilon_{t}^{\left(k\right)}$$ (17)

$$Y_{t}^{\left(m\right)}=\sum_{j=1}^{p}{\hat{A}}_{j}^{\left(m,l\right)}Y_{t-j}^{\left(l\right)}+\sum_{j=1}^{p}{\hat{A}}_{j}^{\left(m,k\right)}Y_{t-j}^{\left(k\right)}+\sum_{j=1}^{p}{\hat{A}}_{j}^{\left(m,m\right)}Y_{t-j}^{\left(m\right)}+\sum_{i=1}^{q}B_{i}^{\left(m\right)}u_{t-i}+\varepsilon_{t}^{\left(m\right)}$$ (18)

Thus, for the above MVARX, the GC that the exogenous input *u*(*t*) exerts in the (group of) region(s) **Y***^i^*, *i* = *l*, *k*, *m* reads:

$$F_{u\to Y^{\left(i\right)}}=\ln\frac{\left|\sum_{e^{i}e^{i}}\right|}{\left|\sum_{\varepsilon^{i}\varepsilon^{i}}\right|},$$ (19)

where $\sum_{e^{i}e^{i}}$ is the covariance of the residuals of the (restricted) MVAR model given by Eq.(9),(10),(11) i.e. the full model but without the exogenous variable *u*.

Thus, if $\left|\sum_{\varepsilon^{i}\varepsilon^{i}}\right|=\left|\sum_{{}_{e^{i}e^{i}}}\right|$ then $F_{u\to Y^{\left(S\right)}}=0$, implying that the input *u* does not excite the brain region(s) **Y***^i^*; if $\left|\sum_{\varepsilon^{i}\varepsilon^{i}}\right|<\left|\sum_{e^{i}e^{i}}\right|$, then $F_{u\to Y^{\left(i\right)}}>0$. By hypothesis, we expect that one of the computed causalities, say $F_{u\to Y^{\left(l\right)}}$ will be significantly larger than the other ones, i.e. $F_{u\to Y^{\left(l\right)}}>>\left\{F_{u\to Y^{\left(k\right)}},F_{u\to Y^{\left(m\right)}}\right\}$.

The above can be straightforwardly generalized to a network with *n* nodes and *m* driving inputs. In that case, for each exogeneous driving input we calculate *n* unidirectional conditional GCs (one for each node) and if, for instance, the *i*-th GC is not zero, we infer that the driving input acts on the *i*-th node (brain region). This analysis reveals which region is driven by a particular driving input.

#### 2.2.2. Assessment of modulatory effects

Having constructed the *emergent* causal connectivity network (from the first stage of the approach) and assessed the effect of the exogenous input *u* (from the second stage of the approach), at the third stage we assess the impact of modulation on the *emergent* interconnections.

**Figure 2.**
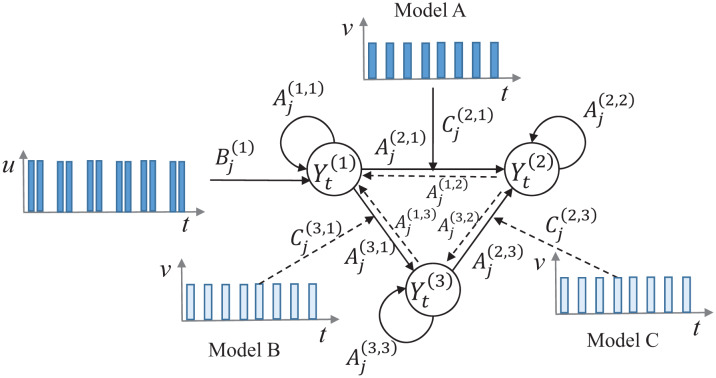
A schematic of a three-node network with an exogenous input *u*, and a modulatory input *v*, which can in principle influence any of the three *emergent* connections between the three nodes. For our demonstrations, we assume that *v* is a repeated signal that modulates one of the three *emergent* connections at every time instance.

For our illustrations, let us consider one exogenous stimulus (*u*) and a modulatory input (*v*) acting on an *emergent* network involving three (brain) regions (see Figure 2) whose activity is represented by the time series **Y**^(1)^, **Y**^(2)^ and **Y**^(2)^. In general, the modulatory input can influence any of the three *emergent* connections between the three regions. For the configuration shown in Figure (2), the modulatory input influences one of the three *emergent* connections marked with solid lines, there are three candidate MVARX models, which are given below.

*Model A*

$$Y_{t}^{\left(1\right)}=\sum_{j=1}^{p}A_{j}^{\left(1,1\right)}Y_{t-j}^{\left(1\right)}+\sum_{j=1}^{p}A_{j}^{\left(1,2\right)}Y_{t-j}^{\left(2\right)}+\sum_{j=1}^{p}A_{j}^{\left(1,3\right)}Y_{t-j}^{\left(3\right)}+\sum_{j=1}^{q}B_{j}^{\left(1\right)}u_{t-j}+e_{t}^{\left(1\right)}$$ (20)

$$Y_{t}^{\left(2\right)}=\sum_{j=1}^{p}\left(A_{j}^{\left(2,1\right)}+C_{j}^{\left(2,1\right)}v_{t-j}\right)Y_{t-j}^{\left(1\right)}+\sum_{j=1}^{p}A_{j}^{\left(2,2\right)}Y_{t-j}^{\left(2\right)}+\sum_{j=1}^{p}A_{j}^{\left(2,3\right)}Y_{t-j}^{\left(3\right)}+e_{t}^{\left(2\right)}$$ (21)

$$Y_{t}^{\left(3\right)}=\sum_{j=1}^{p}A_{j}^{\left(3,1\right)}Y_{t-j}^{\left(1\right)}+\sum_{j=1}^{p}A_{j}^{\left(3,2\right)}Y_{t-j}^{\left(2\right)}+\sum_{j=1}^{p}A_{j}^{\left(3,3\right)}Y_{t-j}^{\left(3\right)}+e_{t}^{\left(3\right)}$$ (22)

*Model B*

$$Y_{t}^{\left(1\right)}=\sum_{j=1}^{p}A_{j}^{\left(1,1\right)}Y_{t-j}^{\left(1\right)}+\sum_{j=1}^{p}A_{j}^{\left(1,2\right)}Y_{t-j}^{\left(2\right)}+\sum_{j=1}^{p}A_{j}^{\left(1,3\right)}Y_{t-j}^{\left(3\right)}+\sum_{j=1}^{q}B_{j}^{\left(1\right)}u_{t-j}+e_{t}^{\left(1\right)}$$ (23)

$$Y_{t}^{\left(2\right)}=\sum_{j=1}^{p}A_{j}^{\left(2,1\right)}Y_{t-j}^{\left(1\right)}+\sum_{j=1}^{p}A_{j}^{\left(2,2\right)}Y_{t-j}^{\left(2\right)}+\sum_{j=1}^{p}A_{j}^{\left(2,3\right)}Y_{t-j}^{\left(3\right)}+e_{t}^{\left(2\right)}$$ (24)

$$Y_{t}^{\left(3\right)}=\sum_{j=1}^{p}\left(A_{j}^{\left(3,1\right)}+C_{j}^{\left(3,1\right)}v_{t-j}\right)Y_{t-j}^{\left(1\right)}+\sum_{j=1}^{p}A_{j}^{\left(3,2\right)}Y_{t-j}^{\left(2\right)}+\sum_{j=1}^{p}A_{j}^{\left(3,3\right)}Y_{t-j}^{\left(3\right)}+e_{t}^{\left(3\right)}$$ (25)

*Model C*

$$Y_{t}^{\left(1\right)}=\sum_{j=1}^{p}A_{j}^{\left(1,1\right)}Y_{t-j}^{\left(1\right)}+\sum_{j=1}^{p}A_{j}^{\left(1,2\right)}Y_{t-j}^{\left(2\right)}+\sum_{j=1}^{p}A_{j}^{\left(1,3\right)}Y_{t-j}^{\left(3\right)}+\sum_{j=1}^{q}B_{j}^{\left(1\right)}u_{t-j}+e_{t}^{\left(1\right)}$$ (26)

$$Y_{t}^{\left(2\right)}=\sum_{j=1}^{p}A_{j}^{\left(2,1\right)}Y_{t-j}^{\left(1\right)}+\sum_{j=1}^{p}A_{j}^{\left(2,2\right)}Y_{t-j}^{\left(2\right)}+\sum_{j=1}^{p}\left(A_{j}^{\left(2,3\right)}+C_{j}^{\left(2,3\right)}v_{t-j}\right)Y_{t-j}^{\left(3\right)}+e_{t}^{\left(2\right)}$$ (27)

$$Y_{t}^{\left(3\right)}=\sum_{j=1}^{p}A_{j}^{\left(3,1\right)}+Y_{t-j}^{\left(1\right)}+\sum_{j=1}^{p}A_{j}^{\left(3,2\right)}Y_{t-j}^{\left(2\right)}+\sum_{j=1}^{p}A_{j}^{\left(3,3\right)}Y_{t-j}^{\left(3\right)}+e_{t}^{\left(3\right)}$$ (28)

Some of the regression coefficients in the above model depend on the modulatory input *v_t_*.

Now, each one of the equations that depend on the modulatory input (e.g. Eq. (21) in Model A) can be re-written as:

$$Y_{t}^{\left(2\right)}=\sum_{j=1}^{p}A_{j}^{\left(2,1\right)}Y_{t-j}^{\left(1\right)}+\sum_{j=1}^{p}C_{j}^{\left(2,1\right)}Y_{t-j}^{\left(1^{\prime }\right)}+\sum_{j=1}^{p}A_{j}^{\left(2,2\right)}Y_{t-j}^{\left(2\right)}+\sum_{j=1}^{p}A_{j}^{\left(2,3\right)}Y_{t-j}^{\left(3\right)}+e_{t}^{\left(2\right)},$$ (29)

where

$$Y_{t}^{\left(1^{\prime }\right)}=v_{t}Y_{t}^{\left(1\right)}$$ (30)

Thus, by using the above transformation, the modulation of an external input *v*(*t*) is included in a new time series defined by Eq.(30). In a similar manner, Eq. (25) in Model B can be re-written as:

$$Y_{t}^{\left(3\right)}=\sum_{j=1}^{p}A_{j}^{\left(3,1\right)}Y_{t-j}^{\left(1\right)}+\sum_{j=1}^{p}C_{j}^{\left(3,1\right)}Y_{t-j}^{\left(1^{\prime }\right)}+\sum_{j=1}^{p}A_{j}^{\left(3,2\right)}Y_{t-j}^{\left(2\right)}+\sum_{j=1}^{p}A_{j}^{\left(3,3\right)}Y_{t-j}^{\left(3\right)}+e_{t}^{\left(3\right)},$$ (31)

where,

$$Y_{t}^{\left(1^{\prime }\right)}=v_{t}Y_{t}^{\left(1\right)}$$ (32)

Finally, Eq. 27 in Model C can be re-written as:

$$Y_{t}^{\left(2\right)}=\sum_{j=1}^{p}A_{j}^{\left(2,1\right)}Y_{t-j}^{\left(1\right)}+\sum_{j=1}^{p}A_{j}^{\left(2,2\right)}Y_{t-j}^{\left(2\right)}+\sum_{j=1}^{p}A_{j}^{\left(2,3\right)}Y_{t-j}^{\left(3\right)}+\sum_{j=1}^{p}C_{j}^{\left(2,3\right)}Y_{t-j}^{\left(3^{\prime }\right)}+e_{t}^{\left(2\right)},$$ (33)

where

$$Y_{t}^{\left(3^{\prime }\right)}=v_{t}Y_{t}^{\left(3\right)}$$ (34)

Using the above formulation, we can now compute the corresponding measures for each one of the above models on the basis of the causalities that the new time series ***Y***^(*i*′)^ exert to the original time series ***Y***^(*i*)^.

For the above configuration the corresponding conditional GC (e.g. for Model A) reads:

$$F_{Y^{\left(1^{\prime }\right)}\to Y^{\left(2\right)}|Y^{\left(1\right)},Y^{\left(2\right)},Y^{\left(3\right)}}=ln\frac{\left|\sum_{{}_{e^{\left(2^{\prime }\right)}e^{\left(2^{\prime }\right)}}}\right|}{\left|\sum_{e^{\left(2\right)}e^{\left(2\right)}}\right|},$$ (35)

where $\sum_{e^{2^{\prime }}e^{2^{\prime }}}$ is the estimator of the covariance of the residuals of the restricted model of Eq. (29), (30) given by:

$$Y_{t}^{\left(2\right)}=\sum_{j=1}^{p}A_{j}^{\left(2,1\right)}Y_{t-j}^{\left(1\right)}+\sum_{j=1}^{p}A_{j}^{\left(2,2\right)}Y_{t-j}^{\left(2\right)}+\sum_{j=1}^{p}A_{j}^{\left(2,3\right)}Y_{t-j}^{\left(3\right)}+{e^{\prime }}_{t}^{\left(2\right)},$$ (36)

If $F_{Y^{\left(1^{\prime }\right)}\to Y^{\left(2\right)}|Y^{\left(1\right)},Y^{\left(2\right)},Y^{\left(3\right)}}>0$ it means that when past values of the new time series ***Y****_t_*^(1′)^ = *v_t_**Y***^(1)^ are involved in the full model given by Eq.(29),(30), then the prediction of ***Y***^(2)^ becomes better, rather than using the restricted model given by Eq.(36) which does not takes into account ***Y****_t_*^(1′)^.

In a system with *n* (groups of) brain regions, with one modulatory input *v*, one should estimate all possible combinations that match with the emergent connectivity scheme.

## 3. Examples validating the method and discussion

### 3.1. Synthetic simulations

**Figure 3.**
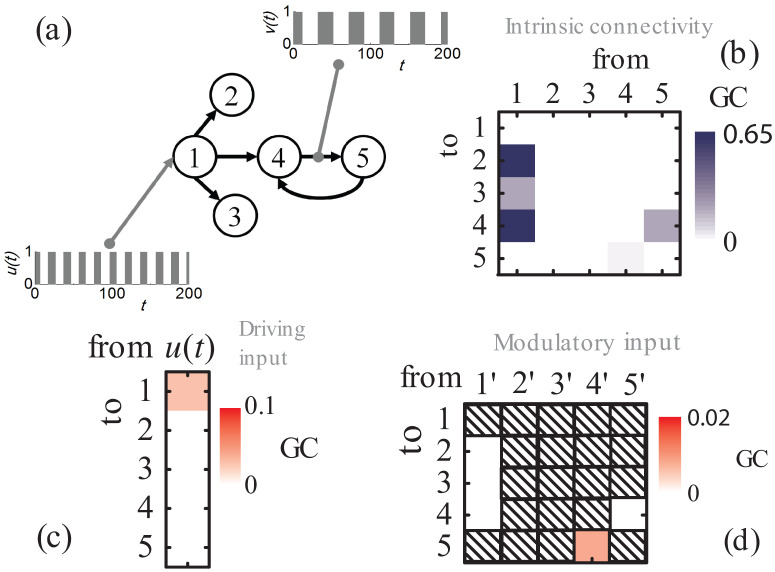
(a) Connectivity diagram of the toy model. (b) Mean values of conditional GC over 100 sets of calculations between the five time series of Eq. (37) assuming significance level *α* = 0.01, which corresponds to the inferred *emergent* connectivity scheme. (c) Averaged conditional GC directed from the driving input *u*(*t*) to each node of the model. (d) Averaged conditional GC directed from each of the modified time series *Y*^(*i*′)^ to the standard *Y*^(*j*)^ (*i*′ ≠ *j*) time series. Since the time series *Y*^(*i*′)^ involves the modulatory input *v*(*t*), this magnitude is a measure of the influence of the modulatory input on the particular unidirectional connection.

First, the performance of the proposed scheme is tested through a five-node network benchmark model (see e.g. Ref. [65]) that is appropriately modified to include a driving/exogenous (*u_t_*) and a modulatory (*v_t_*) input, as shown in Figure 3(a). The equations that generate the synthetic data series are the following:

$$\begin{array}{l} Y_{t}^{\left(1\right)}=0.5u_{t-1}+0.95\sqrt{2}Y_{t-1}^{\left(1\right)}-0.9025Y_{t-2}^{\left(1\right)}+w_{t}^{\left(1\right)}\\ Y_{t}^{\left(2\right)}=0.5Y_{t-2}^{\left(1\right)}+w_{t}^{\left(2\right)}\\ Y_{t}^{\left(3\right)}=-0.4Y_{t-3}^{\left(1\right)}+w_{t}^{\left(3\right)}\\ Y_{t}^{\left(4\right)}=-0.5Y_{t-2}^{\left(1\right)}+0.25\sqrt{2}Y_{t-1}^{\left(4\right)}+0.25\sqrt{2}Y_{t-1}^{\left(5\right)}+w_{t}^{\left(4\right)}\\ Y_{t}^{\left(5\right)}=0.25\sqrt{2}[v_{t-1}-1]Y_{t-1}^{\left(4\right)}+0.25\sqrt{2}Y_{t-1}^{\left(5\right)}+w_{t}^{\left(5\right)}, \end{array}$$ (37)

where, *u_t_* is the driving input and *v_t_* is the modulatory input, respectively. The time series *w_t_*^(*i*)^, *i* = 1,2,3,4,5 are zero-mean, unit standard deviation, uncorrelated normally-distributed noise.

First, we performed a conditional MVGC analysis, using the *Multivariate Granger Causality* (MVGC) toolbox [28], between time series *Y_t_*^(*i*)^, *i* = 1,2,3,4,5, to extract the *emergent* connectivity network. To produce the synthetic data series of Eq. (37) we substituted the genvar.m routine in MVGC toolbox with the genvar– inputs.m routine, which is available in Supplemental Material. We assumed a time step of 1 s and generated 1000 s of data. The first 250 s where discarded to avoid numerical *burn-in* effects. Stationarity tests were performed using the MVGC toolbox, where in all cases the spectral radius of the estimated full VAR model is found less than one. A thorough error-checking, multiple-hypothesis adjustment, and construction of confidence intervals have been also taken care. The model order was estimated using the Akaike Information Criterion (AIC) [27], [28]. The results of the model-order analysis (mean values and standard deviations calculated over 100 independent runs of each multivariate model in each step as described in Section 2.2) are given in Table 1. The results of the conditional MVGC analysis are shown in Figure 3(b)–(d). Under permutation tests using 100 independent runs, the following GCs are found above the statistical-significance threshold set to 0.01: *F*_1→2_ = 0.613, *F*_1→3_ = 0.166, *F*_1→4_ = 0.540, *F*_4→5_ = 0.015, and *F*_5→4_ = 0.134. The corresponding confidence intervals have also been estimated using bootstrap of 100 samples. The confidence intervals are the following: $F_{1\to 2}^{\text{(conf.int.})}=(0.536,0.713)$, $F_{1\to 3}^{\text{(conf.int.})}=(0.118,0.231)$, $F_{1\to 4}^{\left(\text{conf.int.}\right)}=(0.465,0.636)$, $F_{4\to 5}^{\left(\text{conf.int.}\right)}=(0.004,0.041)$, and $F_{5\to 4}^{\text{(conf.int.})}=(0.091,0.194)$, where the first number inside the parenthesis is the lower and the second the upper confidence bound. Obviously, the statistically-significant GCs infer the actual *emergent* connectivity scheme.

**Table 1.** Estimated model orders for the toy model.

Model orders	mean value	stand. deviation
*emergent* connectivity	2.96	0.20
Driving input	2.84	0.37
Modulatory input (1′)	2.67	0.47
Modulatory input (2′)	2.92	0.27
Modulatory input (3′)	2.89	0.31
Modulatory input (4′)	2.74	0.44
Modulatory input (5′)	2.63	0.49

As a next step, we estimated the GC from the driving input *u*(*t*) to each one of the *internal* time series *Y_t_*^(*i*)^, *i* = 1,2,3,4,5. The results of this analysis are presented in Figure 3(c). The analysis revealed only one statistically significant GC: the *F_u_*_→1_ = 0.059, with $F_{u\to 1}^{\left(\text{conf.int.}\right)}=(0.031,0.103)$. This finding is also in agreement with the actual connectivity pattern shown in Figure 3(a).

**Figure 4.**
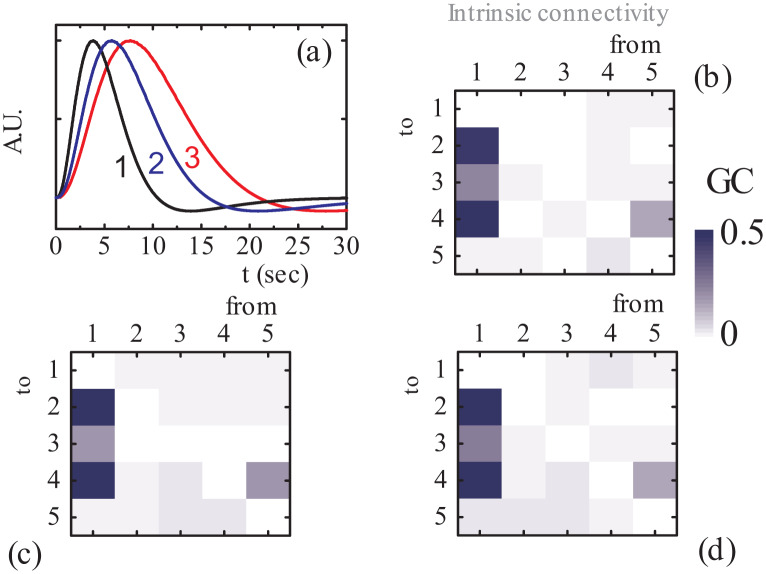
(a) Canonical HRFs with different rise times and shapes. (b), (c), (d) *Emergent* connectivity maps: Conditional GC of the VAR outputs convolved with HRF-1, -2, -3 of (a), respectively.

As a final step, we assessed the influence of the modulatory input *v_t_*. This step includes the generation of new five modified time series *Y_t_*^(*i′*)^ = *v_t_Y_t_*^(*i*)^, *i* = 1,2,3,4,5, one for each of the five (see Figure (4) different *emergent* candidate connections. Thus, we constructed five models, each involving six time series (the five initial time series plus a new modified one, i.e. the *i*′-th, (*i*′ = 1,2,3,4,5)). For each one of the models we performed a MVGC analysis as described in the previous section. The analysis revealed correctly that the only statistically significant GC regarding the modulation is the *F*_4′→5_ = 0.011, with $F_{4^{\prime }\to 5}^{\left(\text{conf.int.}\right)}=(0.002,0.034)$, (see Figure 3(d)). This indicates that the modulatory input *v_t_* influences only the connection from node 4 to node 5.

In brief, the proposed scheme reconstructed successfully the actual connectivity pattern presented in Figure 3(a). The scheme succeeded also to reconstruct the actual networks for different/alternative configurations of the the driving and modulatory inputs.

### 3.2. Influence of haemodynamic latencies

In typical fMRI experiments the measured signals undergo haemodynamic latencies which are found to challenge the subsequent GC analysis [63]. For that reason, we also assessed the efficiency of the proposed analysis by considering haemodynamic latencies in the time series of the toy model of Figure 3(a). Actually, we convoluted the synthetic signals, produced by Eqs. (37), using canonical HRF functions, and then employed the proposed analysis onto the convoluted series; the convoluted time series were imported to MVGC toolbox for GC analysis. The considered HRFs are generated using the values of Ref. [66] which are based on experimentally observed data. As shown in Figure 4(a) the three considered canonical HRFs have different time-to-peak values, i.e., HRF-1 peaks at 3.8 s, while HRF-2 and HRF-3 peak at 5.7 and 7.7 s, respectively.

In these cases, the AIC used so far gives an almost 10-fold increased model order, while, on the other hand, the Bayesian Information Criterion (BIC) gives roughly a 4-fold increment. Consequently, in such, fMRI-like (HRF-convoluted) cases, it is more suitable to adopt the BIC for the estimation of the model order, as indicated also in Refs [27], [29], [63].

As a first step, we performed a GC analysis for the HRF-1 convoluted data set. The estimated model order (mean values and standard deviations) from 100 independent runs of each data set are shown in Table 2. Setting again the significance level to 0.01 (keeping it constant for all subsequent analyses), the permutation significance test based on the theoretical *χ*^2^ asymptotic null distribution [63] resulted in the GCs (see Figure 4(b)): $F_{1\to 2}^{\left(\text{HRF}-1\right)}=0.484$, $F_{1\to 3}^{\text{(HRF}-1)}=0.238$, $F_{1\to 4}^{\text{(HRF}-1)}=0.653$, $F_{4\to 5}^{\text{(HRF}-1)}=0.040$, and $F_{5\to 4}^{\text{(HRF}-1)}=0.168$ which are above the significance threshold. In a similar manner, the GC analysis of the HRF-2-convoluted data set gives the following GCs (see Figure 4(b)): $F_{1\to 2}^{\left(\text{HRF}-2\right)}=0.524$, $F_{1\to 3}^{\left(\text{HRF}-2\right)}=0.200$, $F_{1\to 4}^{\left(\text{HRF}-2\right)}=0.617$, $F_{4\to 5}^{\left(\text{HRF}-2\right)}=0.043$, $F_{5\to 4}^{\text{(HRF}-2)}=0.203$, and also $F_{3\to 4}^{\text{(HRF}-2)}=0.042$, $F_{3\to 5}^{\left(\text{HRF}-2\right)}=0.044$. As we can see, the extracted GCs $F_{3\to 4}^{\left(\text{HRF}-2\right)}$ and $F_{3\to 5}^{\text{(HRF}-2)}$ are spurious, induced from the corresponding haemodynamic latencies. Finally, the convolution with HRF-3 gives the following significant GCs (see also Figure 4(d)): $F_{1\to 2}^{\text{(HRF}-3)}=0.519$, $F_{1\to 3}^{\left(\text{HRF}-3\right)}=0.259$, $F_{1\to 4}^{\left(\text{HRF}-3\right)}=0.656$, and $F_{5\to 4}^{\left(\text{HRF}-3\right)}=0.167$. In the latter case, although the two spurious GCs are missing, a real GC is also missing, i.e., the $F_{4\to 5}^{\left(\text{HRF}-3\right)}$. These findings indicate that, although for the first case (HRF-1) the actual *emergent* connectivity is inferred, as the haemodynamic latencies increase (HRF-2, HRF-3), the inferring capability of the GC analysis is blurred. However, following the findings of Ref. [63], we should have in mind that although hemodynamic filtering hinders the reliability of statistical-significance tests, the calculated GCs magnitudes still carry significant information.

**Table 2.** Model orders for the model with haemodynamic latencies.

Toy model with HRFs	mean value	stand. deviation
*Emergent* connectivity - conv. HRF-1	12.59	0.68
*Emergent* connectivity - conv. HRF-2	17.17	1.01
*Emergent* connectivity - conv. HRF-3	22.02	1.38
–	–	–
Driving input - conv. HRF-1	13.6	1.77
Driving input - conv. HRF-2	20.10	0.33
Driving input - conv. HRF-3	21.61	0.84
–	–	–
Modulatory input (1′) - conv. HRF-1	12.11	0.55
Modulatory input (2′) - conv. HRF-1	11.97	0.50
Modulatory input (3′) - conv. HRF-1	12.06	0.57
Modulatory input (4′) - conv. HRF-1	12.03	0.48
Modulatory input (5′) - conv. HRF-1	12.04	0.37
Modulatory input (1′) - conv. HRF-2	16.48	0.86
Modulatory input (2′) - conv. HRF-2	16.46	0.64
Modulatory input (3′) - conv. HRF-2	16.36	0.60
Modulatory input (4′) - conv. HRF-2	16.40	0.65
Modulatory input (5′) - conv. HRF-2	16.46	0.70
Modulatory input (1′) - conv. HRF-3	21.06	1.50
Modulatory input (2′) - conv. HRF-3	20.76	1.80
Modulatory input (3′) - conv. HRF-3	20.85	1.40
Modulatory input (4′) - conv. HRF-3	20.82	1.55
Modulatory input (5′) - conv. HRF-3	20.77	1.22

**Figure 5.**
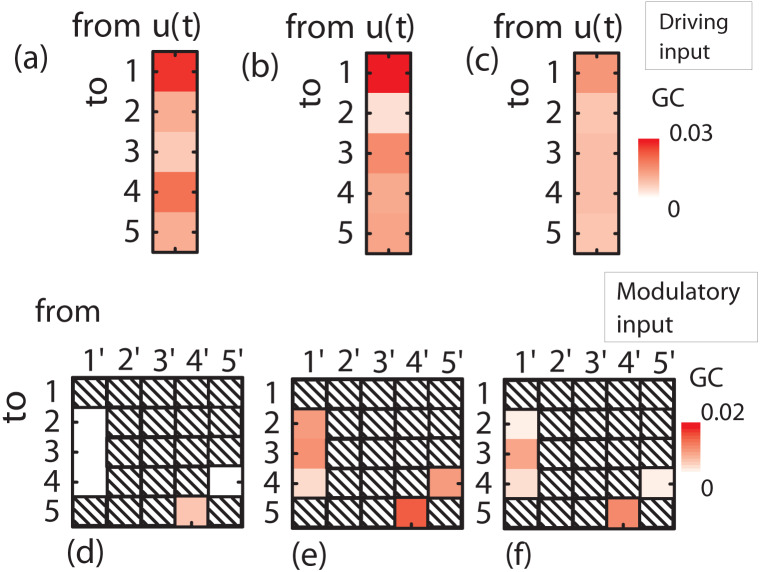
(a)–(c) Averaged conditional GC directed from the driving input *u*(*t*) to each node of the model system: Conditional GC of the VAR outputs convolved with HRF (1)-(3), respectively. (d)–(f) Averaged conditional GC directed from each of the modified time series *Y*^(*i*′)^ to the *Y*^(*i*′)^ time series: Conditional GC of the VAR outputs convolved with HRF (1)-(3), respectively.

As a next step, we applied the proposed scheme to access the impact of haemodynamic convolution with respect to the driving input. The estimated model orders for each case are shown in Table 2. As can be seen in Figure 5(a)–(c) the most prominent GC corresponds again to the actual connectivity pattern, although the haemodynamic convolution blurs the picture. Particularly, when convoluting with HRF-1, the strongest GC is $F_{u\to 1}^{\left(\text{HRF}-1\right)}=0.027$, as shown in Figure 5(a), while the rest GCs are weaker but non-zero. Similarly, convoluting with HRF-2 and HRF-3 the larger GCs are $F_{u\to 1}^{\left(\text{HRF}-2\right)}=0.036$ and $F_{u\to 1}^{\left(\text{HRF}-3\right)}=0.015$, respectively. We see, that, although again the significance assessment is severely hindered, the GC magnitudes can still suggest the actual region (region 1) through which the external driving stimuli enters into the network. Next, the assessment of the external modulation *v* on each connection, for latent time series convoluted with HRF-1 is shown in Figure 5(d). The results predict only one statistically significant GC, i.e., $F_{4^{\prime }\to 5}^{\left(\text{HRF}-1\right)}=0.006$, while the rest GCs are found below the statistical significance level and almost one order of magnitude weaker than *F*_4′→5_. Hence, the actual modulated connection is successfully inferred in this case. When the haemodynamic latencies are increased, i.e., when taking HRF-2 and HRF-3, again *F*_4′→5_ corresponds to the strongest GC, as shown in Figure 5(e–f), indicating an actual underlying mechanism, although the significance testing is undermined, as explained previously. In fact, for HRF-2 we have, $F_{4^{\prime }\to 5}^{\left(\text{HRF}-2\right)}=0.015$ and the other GC magnitudes are below 0.010, while for the HRF-3 convoluted time-series we have, $F_{4^{\prime }\to 5}^{\left(\text{HRF}-3\right)}=0.009$ and the other GC magnitudes are below 0.007. The above findings indicate that the inferring capability of the proposed approach is sufficiently preserved for moderate haemodynamic latencies.

### 3.3. Application to experimental task-related fMRI data: the attention to visual motion benchmark problem

The role of the attention in the perception of visual motion has been extensively studied using fMRI [67], [68]. In their landmark work, C. Buchel and K. Friston [67] using DCM showed that attention modulates the connectivity between brain regions involved in the perception of visual motion. In the present work, we analyzed the same fMRI dataset ([67]) which has served as benchmark problem in many studies [30], [54], [69]. The dataset was downloaded from the official SPM website (http://www.fil.ion.ucl.ac.uk/spm/data/attention/), where the fMRI data are smoothed, spatially normalized, realigned, and slice-time corrected. As described in Ref. [67], in the original experiment the subjects were observing a black computer screen displaying white dots. According to the experimental design, there where specific epochs, where the dots where either static or moving. Intermediate epochs without dots and only one static picture were also introduced. In several epochs of moving dots, the subjects were instructed to keen on possible changes in the velocity of the moving dots, although no changes really existed. Therefore, three experimental variables are considered: *photic* for visual stimulation, *motion* for moving dots, and *attention* for the observation of possible changes in the velocity of the dots. Following Refs. [67]–[69], we identified three activated brain regions, namely the V1, V5 and SPC, and extracted the corresponding time series through eigendecomposition [67], using the Statistical Parametric Mapping toolbox (SPM12) [70]–[72]. In previous works, the aforementioned connectivity network had been intensively investigated using the DCM method [54], [69].

**Figure 6.**
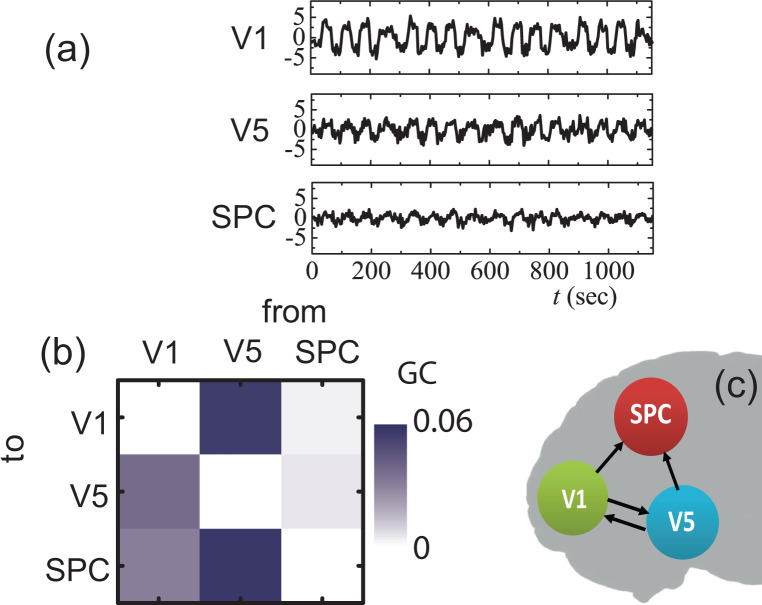
(a) fMRI time series that correspond to brain regions V1, V5 and SPC, respectively. (b) Estimated *emergent* GCs, for model order equal to 1. (c) A schematic of the inferred functional connectivity network from (b).

Here, we applied, the proposed extended GC analysis, to infer the underlying connectivity network. The extracted time series for each brain region involved in this task are shown in Figure 6(a). By importing the three extracted time series (V1, V5 and SPC) into the MVGC toolbox, we obtained the emerging connectivity matrix shown in Figure 6(b).

The model order, according to BIC is 1, which can be attributed to the low sampling rate of the particular fMRI data [29], [63]. The estimated non-zero (with a significance level set at 0.01) GCs using a bootstrap of 100 samples are the following: *F*_V1→V5_ = 0.038 with $F_{\text{V}1\to \text{V}5}^{\left(\text{conf.int.}\right)}=(0.005,0.099)$, *F*_V1→SPC_ = 0.031 with $F_{\text{V1}\to \text{SPC}}^{\left(\text{conf.int.}\right)}=(0.003,0.088)$, *F*_V5→V1_ = 0.056 with $F_{\text{V5}\to \text{V1}}^{\left(\text{conf.int.}\right)}=(0.014,0.127)$, *F*_V5→SPC_ = 0.058 with $F_{\text{V5}\to \text{SPC}}^{\left(\text{conf.int.}\right)}=(0.015,0.130)$. According to the above results, a visualization of the resulted inferred functional connectivity network is shown in Figure 6(c).

**Figure 7.**
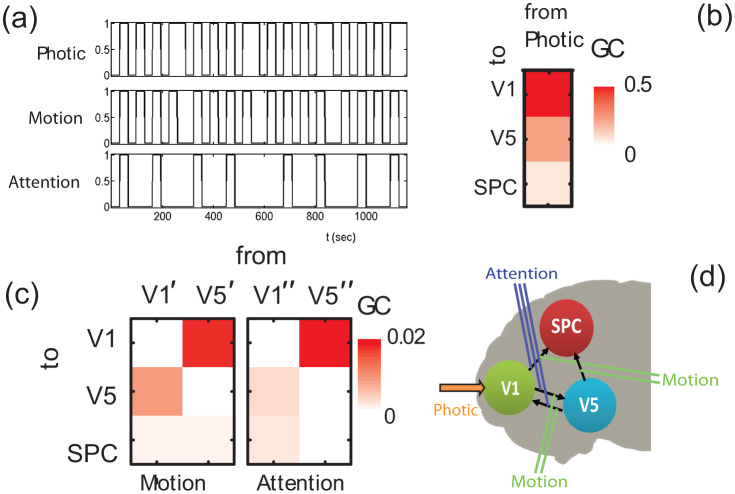
(a) Input time series for Photic, Motion and Attention respectively. (b) Extracted GCs from the Photic time series to each node time series. (c) Extracted modulation by motion and attention, respectively. (d) Inferred connectivity scheme, including also the external inputs.

As a next step, we sought to associate the external visual stimuli (driving input) with one of the brain regions involved. In this respect, we estimated the causal effect of the Photic time series, shown in Figure 7(a), on the V1, V5, and SPC time series. For this reason, we implemented the proposed extended conditional MVGC analysis on a 4-variable system; we imported the three time series of Figure 6(a), i.e., V1, V5 and SPC, together with the Photic time series of Figure 7(a) into the MVGC toolbox and performed the GC analysis. The results are shown in Figure 7(b), where *F*_Photic→V1_ = 0.450 with $F_{\text{Photic}\to \text{V}1}^{\text{(conf.int.})}=(0.326,0.594)$, *F*_Photic→V5_ = 0.209 with $F_{\text{Photic}\to \text{V}2}^{\left(\text{conf.int.}\right)}=(0.120,0.323)$, and *F*_Photic→SPC_ = 0.051 with $F_{\text{Photic}\to \text{SPC}}^{\text{(conf.int.})}=(0.011,0.120)$. All GC magnitudes are found statistically significant. However, since *F*_Photic→V1_ is obviously predominant, we can assume that the external visual stimulation affects more the V1 region and subsequently, through V1, it is transferred to the regions V5 and SPC.

Finally, to complete the circuit diagram, we assessed the modulatory effect of two inputs, namely that of motion and attention, whose time series are shown in Figure 7(a). Since we have two modulatory inputs, we constructed two modified time series that correspond to Motion stimuli, denoted with ′ and to Attention stimuli denoted with ″. The results of the proposed analysis are shown in Figure 7(c). We computed all possible causal flows which, for the Motion resulted to *F*_V1′→V5_ = 0.010 with $F_{\text{V}1^{\prime }\to \text{V}5}^{\left(\text{conf.int.}\right)}=(0.000,0.049)$, *F*_V1′→SPC_ = 0.002 with $F_{\text{V}1^{\prime }\to \text{SPC}}^{\text{(conf.int.)}}=(0.000,0.026)$ *F*_V5′→V1_ = 0.018 with $F_{\text{V}5^{\prime }\to \text{V}1}^{\left(\text{conf.int.}\right)}=(0.000,0.066)$, *F*_V5′→SPC_ = 0.002 with $F_{\text{V}5^{\prime }\to \text{SPC}}^{\left(\text{conf.int.}\right)}=(0.000,0.027)$, while for the Attention, we got: *F*_V1″→V5_ = 0.004 with $F_{\text{V}1^{\prime \prime }\to \text{V}5}^{\left(\text{conf.int.}\right)}=(0.000,0.034)$, *F*_V1″→SPC_ = 0.003 with $F_{\text{V}1^{\prime \prime }\to \text{SPC}}^{\left(\text{conf.int.}\right)}=(0.000,0.031)$ *F*_V5″→V1_ = 0.022 with $F_{\text{V}5^{\prime \prime }\to \text{V}1}^{\left(\text{conf.int.}\right)}=(0.001,0.074)$, *F*_V5″→SPC_ = 0.000 with $F_{\text{V}5^{\prime \prime }\to \text{SPC}}^{\left(\text{conf.int.}\right)}=(0.000,0.019)$. Thus, the inferred connectivity pattern with the GC modulatory connections is shown in Figure 7(d).

#### 3.3.1. Model comparison using the DCM

**Figure 8.**
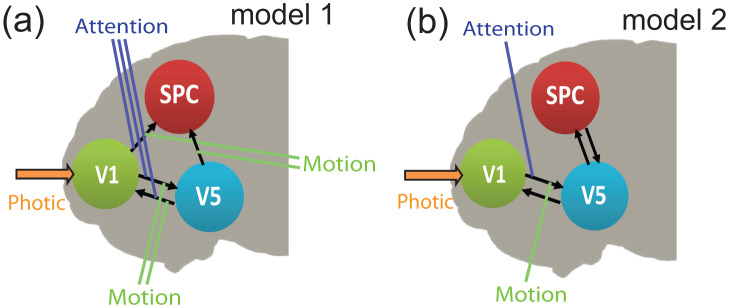
Connectivity analysis of the attention to visual motion fMRI dataset. (a) Connectivity pattern inferred from the proposed fully data-driven GC approach (model 1). (b) Connectivity pattern selected among other candidate models with the DCM based on bayesian analysis (see Refs. [54], [69]) (model 2).

Finally, in order to assess the validity of the resulted network, we compared the inferred model (shown again in Figure 8(a) (model 1)) with an alternative model (model 2) taken from Refs. [54], [69]. In particular, model 2, shown in Figure 8(b) is the reference selected model, obtained in Refs. [54], [69] as the best among other candidate models using DCM analysis. The two models differ with respect to the *emergent* connections between regions V1 and SPC, and between V5 and SPC. Furthermore, model 1 assumes that the modulatory inputs affect more connections. Here, we compared their posterior likelihood through bayesian model selection by importing the two models into the SPM12 software [70]–[72]. The log-evidence (see Ref. [54] for details) for model 1 is found −3221, while for model 2 equals to −3285. Therefore, the natural logarithm of the Bayes factor *B*_12_ equals to 64 suggesting that the data favour model 1 over model 2. We note here, that the net computational time for deducing model 1 using the proposed GC analysis, is less than five seconds, using an i7 processor and 32 GiB RAM. On the other hand, model 2 was taken from Refs. [54], [69] ready-made. However, as detailed in Refs. [54], [69], deducing model 2 requires a prior knowledge, and then, a Bayesian comparison is applied between pairs of alternative models. Since each Bayesian comparison requires one-minute computational time using the aforementioned machine, it comes out that the respective cost is dramatically increased compared to that of GC inference, especially when all possible configurations are included, as shown also in Ref. [73] for typical GC and dynamic-Byesian inference in larger networks.

## 4. Conclusions

Granger causality (GC) and Dynamic causal modelling (DCM) are the two key methodologies for the reconstruction of directed connectivity patterns of brain functioning. GC is considered as a generic data-driven approach that is mostly used for the reconstruction of the *emergent* functional connectivity (brain regions interconnected in terms of statistical dependence) without dealing with the modelling and influence of exogenous and/or modulatory inputs on the network structure. On the other hand, DCM has been mostly used to infer the effective connectivity from task-related fMRI data enabling the selection of specific models (network structures). For a critical discussion on the analysis of connectivity with GC and DCM one can refer to Friston et al. [3]. One of the main points raised in this paper is that GC and DCM are complementary and that GC can in principle be used to provide candidate models for DCM, thus enhancing our ability to better model and understand cognitive integration. However, to date, only very few studies have investigated this possibility. For example, Bajaj et al. [59] compared the performance of GC and DCM using both synthetic and real *resting-state* fMRI data. They found that both methods result to consistent directed functional and effective connectivity patterns. Here, we proposed an extension of the conditional MVGC to deal with *task-related* fMRI data with exogenous/driving and modulatory inputs. Based on both synthetic and real task-related fMRI data, we showed that the proposed GC scheme successfully identifies the functional connectivity patterns. We also show that the computational time that is needed with the proposed scheme is much less compared to DCM. The key-stones of two methods, i.e. their origin and the results that are obtained based on their use are different. Here, we summarize the pros and cons of the two approaches. GC stems out the theory of time-series analysis, and specifically of the model identification of MVAR models, and it is used to provide directed functional connectivity, that is statistical dependence between neuronal systems based on various neuroimaging techniques (mainly EEG, but also MEG and fMRI). On the other hand, DCM is based on a bio-physical modelling approach, targeting at revealing the effective direct connectivity between brain regions from fMRI data. GC has been mostly used until now to detect the *emergent* functional connectivity networks, i.e. the functional conenctivity networks that emerge due to inputs but without modelling them. Our approach aims to resolve this issue. On the other hand, DCM handles explicitly such information. GC offers a general black-box framework, which is relatively computational cheap when dealing with small to medium dimension of data, while DCM is a framework that is based more on a-priori knowledge about the neuronal system under study and finds the best models from a set of plausible model using hypothesis testing and is more computational demanding as it is based on realistic biophysical modelling. Thus, differences in the connectivity networks found by the two methods should be attributed to the above different origins and capabilities. For a detailed review of the pros and cons of GC and DCM one can refer to the paper of Friston [3]. All in all, GC and DCM should not be considered as two *opponent* approaches, but rather as complementary. Thus, our results suggest that the proposed GC scheme may be used as stand-alone and/or complementary approach to DCM to provide new candidate models for DCM for furthe analysis and comparisons.
